# A phase II trial of autologous dendritic cell vaccination and radiochemotherapy following fluorescence-guided surgery in newly diagnosed glioblastoma patients

**DOI:** 10.1186/s12967-017-1202-z

**Published:** 2017-05-12

**Authors:** Susana Inogés, Sonia Tejada, Ascensión López-Díaz de Cerio, Jaime Gállego Pérez-Larraya, Jaime Espinós, Miguel Angel Idoate, Pablo Daniel Domínguez, Reyes García de Eulate, Javier Aristu, Maurizio Bendandi, Fernando Pastor, Marta Alonso, Enrique Andreu, Felipe Prósper Cardoso, Ricardo Díez Valle

**Affiliations:** 1 Cell Therapy Area, Clínica Universidad de Navarra, Avenida Pio XII 36, 31008 Pamplona, Navarra Spain; 2 Immunology and Immunotherapy Department, Clínica Universidad de Navarra, Avenida Pio XII 36, 31008 Pamplona, Navarra Spain; 3 Neurosurgery Department, Clínica Universidad de Navarra, Avenida Pio XII 36, 31008 Pamplona, Navarra Spain; 4 Neurology Department, Clínica Universidad de Navarra, Avenida Pio XII 36, 31008 Pamplona, Navarra Spain; 5 Oncology Department, Clínica Universidad de Navarra, Avenida Pio XII 36, 31008 Pamplona, Navarra Spain; 6 Pathology Department, Clínica Universidad de Navarra, Avenida Pio XII 36, 31008 Pamplona, Navarra Spain; 7 Radiology Department, Clínica Universidad de Navarra, Avenida Pio XII 36, 31008 Pamplona, Navarra Spain; 8 Radiation Oncology Department, Clínica Universidad de Navarra, Avenida Pio XII 36, 31008 Pamplona, Navarra Spain; 9 Section on Hematology/Oncology, Department of Internal Medicine, Comprehensive Cancer Center, Wake Forest University Baptist Healthcare Center, Winston-Salem, NC USA; 10 Section of Hematology/Oncology, Department of Internal Medicine, W.G Hefner VA Medical Center, Salisbury/Charlotte, NC USA; 11 Program of Molecular Therapies, Aptamer Unit, Centro de Investigación Médica Aplicada (CIMA), Universidad de Navarra, Avenida Pio XII 55, 31008 Pamplona, Navarra Spain; 12 Program in Solid Tumors and Biomarkers, Centro de Investigación Médica Aplicada (CIMA), Universidad de Navarra, Avenida Pio XII 55, 31008 Pamplona, Navarra Spain; 13 Haematology and Haemotherapy Department, Clínica Universidad de Navarra, Avenida Pio XII 36, 31008 Pamplona, Navarra Spain

**Keywords:** Glioblastoma, Immunotherapy, Dendritic cell, Overall survival

## Abstract

**Background:**

Prognosis of patients with glioblastoma multiforme (GBM) remains dismal, with median overall survival (OS) of about 15 months. It is therefore crucial to search alternative strategies that improve these results obtained with conventional treatments. In this context, immunotherapy seems to be a promising therapeutic option. We hypothesized that the addition of tumor lysate-pulsed autologous dendritic cells (DCs) vaccination to maximal safe resection followed by radiotherapy and concomitant and adjuvant temozolomide could improve patients’ survival.

**Methods:**

We conducted a phase-II clinical trial of autologous DCs vaccination in patients with newly diagnosed patients GBM who were candidates to complete or near complete resection. Candidates were finally included if residual tumor volume was lower than 1 cc on postoperative radiological examination. Autologous DCs were generated from peripheral blood monocytes and pulsed with autologous whole tumor lysate. The vaccination calendar started before radiotherapy and was continued during adjuvant chemotherapy. Progression free survival (PFS) and OS were analyzed with the Kaplan–Meier method. Immune response were assessed in blood samples obtained before each vaccines.

**Results:**

Thirty-two consecutive patients were screened, one of which was a screening failure due to insufficient resection. Median age was 61 years (range 42–70). Karnofsky performance score (KPS) was 90–100 in 29%, 80 in 35.5% and 60–70 in 35.5% of cases. MGMT (O^6^-methylguanine-DNA-methyltransferase) promoter was methylated in 45.2% of patients. No severe adverse effects related to immunotherapy were registered. Median PFS was 12.7 months (CI 95% 7–16) and median OS was 23.4 months (95% CI 16–33.1). Increase in post-vaccination tumor specific immune response after vaccines (proliferation or cytokine production) was detected in 11/27 evaluated patients. No correlation between immune response and survival was found.

**Conclusions:**

Our results suggest that the addition of tumor lysate-pulsed autologous DCs vaccination to tumor resection and combined radio-chemotherapy is feasible and safe. A multicenter randomized clinical trial is warranted to evaluate the potential survival benefit of this therapeutic approach.

*Trial registration* This phase-II trial was registered as EudraCT: 2009-009879-35 and ClinicalTrials.gov Identifier: NCT01006044 retrospectively registered

**Electronic supplementary material:**

The online version of this article (doi:10.1186/s12967-017-1202-z) contains supplementary material, which is available to authorized users.

## Background

Despite multimodal treatment, the prognosis of patients with newly diagnosed glioblastoma (GBM) remains dismal with median OS times of about 15–17 months. [1–3]. RPA classification is based on pre-treatment prognostic factors and stratifies patients in three classes (III, IV and V). In a large historic database including patients not receiving adjuvant temozolomide, OS was found to be 16.3, 11.3 and 6.7 months for classes III, IV and V, respectively [4]. The poor prognosis of these patients [5] compels us to look for new therapeutic strategies.

Fluorescence-guided surgery (FGS) using 5-aminolevulinic (5-ALA) is a technical advance in GBM surgery that has increased the rate of patients who achieve complete radiological resection of the tumor (CR) [6]. This rate used to be less than 40% in excellence centers [7] and is now as high as 83% [8–10]. CR has been proposed as a key factor for potentially successful adjuvant therapies [11].

Several immunotherapy strategies based on dendritic cell vaccines have been attempted in GBM and shown to be safe and tolerable. The results of published phase I/II trials have hinted at efficacy, but the designs of these studies included a high proportion of cases with better prognostic features [12–14]. Therefore, it is still unclear whether immunotherapy can be ultimately beneficial to GBM patients and, if so, to what extent.

We conducted a phase II trial for patients with newly diagnosed GBM based on immunotherapy with ex vivo, tumor lysate-pulsed, autologous DCs following FGS and combined it with radio-chemotherapy with temozolomide (TMZ). We hypothesized that the addition of tumor lysate-pulsed autologous DCs vaccination to maximal safe resection followed by combined radio-chemotherapy with TMZ could improve patients’ survival.

## Methods

### Clinical trial

The clinical trial is a Phase II Clinical Trial to Evaluate Safety and Efficacy of Autologous Dendritic Cell Vaccination in GBM Patients after Complete Surgical Resection using 5-ALA. The study was approved by the Ethics Committee of Navarra and registered with ClinicalTrials.gov (NCT01006044) and EudraCT (2009-009879-35). The Primary Endpoint was evaluation of the treatment impact on progression-free survival.

### Patient population

Consecutive patients aged 18–70 years, who were candidates for total resection of newly diagnosed GBM, were screened. Eligible patients should have not received previous treatment with chemotherapy nor radiotherapy, and resection surgery with less than 1 cm^3^ residual tumor on early postoperative (<72 h) Magnetic Resonance Imaging (MRI) was needed to confirm inclusion. All patients provided written consent. Inclusion and exclusion criteria are detailed in Table 1, and patients’ characteristics are described in Table 2.

**Table 1:** Inclusion and exclusion criteria

Inclusion criteria	Exclusion criteria
Patients with histologically confirmed glioblastoma without having been previously treated with chemotherapy or radiotherapy	Participation in another clinical trial. If the patient has previously participated in another clinical trial, he should wait some time determined by the investigator
Ability to provide informed consent and express their desire to fulfill all protocol requirements during the study period	Patients diagnosed with other malignancies except basal cell carcinoma or scaly skin, cervical carcinoma in situ adequately treated or other tumors treated curatively without recurrence for 3 or more years
Age between 18 and 70 years	Pregnant or lactating women
In case of women of childbearing age, negative pregnancy test	Patients who require immunosuppressive medication
The patient should, in the investigator’s opinion, be able to comply with all clinical trial requirements	Positive serology for HIV, hepatitis B (HBsAg) or hepatitis C
Complete tumor resection surgery guided by fluorescence microscope and 5-ALA, verified by postoperative MRI. It is defined as residual injury captante contrast zero or less than 1 cm^3^	Inability to produce enough material for a minimum of 6 cell vaccines
Availability of sufficient tumor tissue processed under controlled conditions to develop cellular vaccines	Absolute contraindication for the remaining standard treatments glioblastoma (surgery, radiotherapy and chemotherapy)

**Table 2:** Characteristics of the patients included

Case	Gender	Age	KPS	RPA	MGMT	EOR (%)	OS (months)	MMSE	Second-therapy
1	M	69	70	5	Met	100	27.0	28	Tmz
2	M	70	70	5	NoMet	99.3	9.1	27	Ir-Bev
3	F	50	80	5	Met	97.9	66	26	Ir-Bev
4	F	67	60	5	Met	100	27.4	28	Ir-Bev
5	F	70	90	4	Met	100	40.3	30	Ir-Bev
6	F	44	100	3	unMet	100	23.4	30	Ir-Bev
7	F	67	90	5	Met	100	51.4	30	No
8	M	65	70	5	Met	100	3.5*	26	No
9	F	54	70	5	unMet	100	16.8	26	Ir-Bev
10	F	63	90	4	unMet	100	6.1	30	No
11	M	69	80	5	unMet	100	33	26	Ir-Bev
12	M	49	80	4	unMet	100	45.4	28	Ir-Bev
13	M	49	80	4	Met	99.5	51.4	30	Ir-Bev
14	M	47	80	4	Met	99.4	16	25	Ir-Bev
15	F	60	90	4	unMet	100	15	26	Ir-Bev
16	M	58	60	5	unMet	100	23.3	22	Ir-Bev
17	F	57	70	5	unMet	99.2	29.5	25	Ir-Bev
18	F	42	90	3	unMet	100	36.9	28	Ir-Bev
19	F	63	70	5	unMet	100	28.1	19	Ir-Bev
20	M	55	80	4	Met	100	44.7	30	Ir-Bev
21	M	68	70	5	unMet	97.2	13.2	26	No
22	F	44	80	4	unMet	100	38.1	26	Ir-Bev
23	M	65	70	5	unMet	100	7	27	No
24	F	57	80	5	unMet	100	19	29	Ir-Bev
25	M	46	100	3	Met	100	>62.2	30	Tmz
26	M	61	70	5	unMet	100	5.6	15	No
27	F	59	80	4	Met	100	>59.5	30	No
28	F	69	90	4	unMet	100	14.4	28	No
29	M	62	80	4	Met	100	21.4	29	No
30	M	64	90	4	Met	100	9.7*	24	No
31	M	66	80	4	Met	100	22.4	29	Ir-Bev

*KPS* Karnofsky Performace Status, *RPA* recursive partitioning analysis class, *MGMT* methyl-guanine-methyl-transferase, *Met* methylated promoter, *unMet* unmethylated promoter, *EOR* extend of resection, *MMSE* minimental state examination, *OS* overall survival, *Tmz* temozolomide, *Ir* irinotecan, *Bev* bevacizumab

* These patients did not get the vaccination

### Surgery

FGS was performed as previously described with the target of resection of the contrast-enhancing tumor [6, 8]. After surgery, steroids were tapered and discontinued within a few days.

### Pathological evaluation

All samples were evaluated by the same neuropathologist on the basis of the 2007 WHO Classification criteria [15]. The MGMT promoter methylation status was assessed by polymerase chain reaction. P53 and IDH1/2 mutation status was not assessed.

### Vaccine production

Fresh tumor was sent from the operating room to the Cell Therapy Laboratory. Tumor single-cell suspensions were obtained by mechanical disaggregation and then frozen and stored. Tumor lysate was obtained through four cycles of thawing and freezing and then irradiated and stored at −20 °C. Seven days after dexamethasone termination, peripheral blood mononuclear cells were collected by leukapheresis. The procedures involved in the production of the autologous DCs-based, customized vaccines have been described in detail elsewhere [16]. CD14+ cells were selected by immunomagnetic separation using a CliniMacs™ (Miltenyi Biotec, Bergisch Gladbach, Germany) following manufacturer’s instruction. These cells were cultured at 2 × 10^6^ cells/ml in AIM-V (Gibco, Grand Island NY 14072) supplemented with antibiotics, 1000 UI/ml of IL-4 (R&D Systems, Minneapolis) and 1000 UI/ml GM-CSF (Leukine, Genzyme Corporation, Bayer Healthcare, Seattle, WA, USA) in culture bags (Cellgenix, Gaithersburg, MD 20877) at 37 °C in a humidified incubator. IL-4 (500 UI/ml) and GM-CSF (500 UI/ml) were further added to the medium on the 4th day and cultured cells were harvested on the 7th day. These immature DC were adjusted at 10^7^ cells/ml and pulsed with autologous tumor lysate (median 69.82 μg/ml, rank 27.9–75 μg/ml) during 2 h at 37 °C and 5% CO2. At that time, to induce DC maturation, 50 ng/ml of TNF-α (Beromun, Boehringer Ingelheim, España), 1000 UI/ml of IFN-α (Intron A, Schering Corporation, Kenilworth, NJ, USA) and 20 ng/ml Poli I:C (Amersham, GE Healthcare) were added to the medium and cells were placed in culture bags at 2 × 10^6^ cells/ml. Mature DC were harvested on the 8th day and frozen in aliquots following standard procedures until use. Briefly, the cells were resuspended in RPMI-1640 complete medium (500 ml RPMI-1640 (GIBCO, Life Technology) + 50 ml of 10% FCS + 5 ml of l-Glutamine 200 mM (GIBCO, Life Technology) + 5 ml Pen/Strep solution (solution with 10,000 U/ml Pen, 10 mg/ml Strep, GIBCO, Life Technology) at twice the desired cryopreservation concentration. The cryopreservation solution was prepared containing 40% complete RPMI-1640, 40% FCS and 20% DMSO. The cryopreservation vials were placed in the cryopreservation box (5100 Crio 1° Freezing Container, Nalgene) and 500 microliters of the cell suspension were added to each vial; then 500 μl of the cryopreservation solution were added and the final suspension was carefully mixed. The cryopreservation box was brought to a −80 °C freezer and after 24 h the cell vials were stored in a liquid nitrogen tank. Ten million cells were considered the optimal dose for each administration. The viability of cells was determined before and after freezing.

### Treatment schedule

**Fig. 1:**
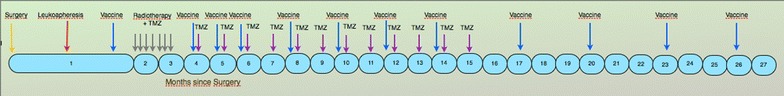
Treatment schedule. All patients were scheduled to receive conventional radio-chemotherapy with up to 12 cycles of adjuvant temozolomide or until disease progression. The first intradermal DCs administrations were scheduled prior to radiotherapy, and the second 3 weeks after radiotherapy. This was followed by two monthly, four bi-monthly, and subsequent quarterly administrations until the end of all available doses. During adjuvant TMZ treatment, DC were administered on day 21 of the corresponding cycle. Vaccines were administered intradermally

All patients were scheduled to receive postoperative intensity modulated radiotherapy with concomitant TMZ (75 mg/m^2^/day), followed by up to 12 cycles of adjuvant TMZ (200 mg/m^2^/day for 5 consecutive days every 28 days) or until disease progression. The first intradermal DCs administrations were scheduled prior to radiotherapy, and the second 3 weeks after radiotherapy. This was followed by two monthly, four bi-monthly, and subsequent quarterly administrations until the end of all available doses (Fig. 1). During adjuvant TMZ treatment, DC were administered on day 21 of the corresponding cycle. Vaccines were administered intradermally, with patients receiving, on average, 8 vaccines. When tumor progression occurred (in 25 of 30 patients), patients were treated at the investigator’s discretion, with the option to maintain vaccination combined with second-line treatment.

### Clinical assessment

Clinical follow-up was carried out monthly during the first year and every other month thereafter. MRI was performed postoperatively (within 72 h after surgery) and every 3 months thereafter. Macdonald criteria were used for response assessment [17]. When progression was suspected, the type of second therapy was irinotecan plus bevacizumab or TMZ, left at the discretion of the treating specialist, with the option to maintain vaccination. All patients were followed until death or until May 2016.

### Immune response assessment

For the immune response evaluation, blood samples were obtained before each vaccine was administered. Mononuclear cells (PBMCs) and serum samples were cryopreserved and thawed together for the assessment. PBMCs were used to analyze tumor specific cellular immune response and serum samples to evaluate changes in cytokines profiles after vaccination.

Tumor-specific cellular immune response were assessed by three methods: T cell proliferation assay, IFN-γ production by enzyme-linked immunosorbent assay (ELISA) and number of IFN-γ producing cells by IFN-γ enzyme-linked immunospot or ELISPOT. Briefly, PBMCs obtained before and after vaccination were plated in 96-well plates at 2 × 10^5^ cells/well with culture medium (RPMI 1640 supplemented with 10% human serum AB, 2 mM glutamine, 100 UI/ml penicillin and 100 μg/ml streptomycin) alone, with 20,000 patient’s tumor lysate-pulsed DC or with 10 μg/ml of lysate tumor only in the case of patients in which DCs are not available. Supernatants were collected after 5 days of culture to measure IFN-γ production. Then, cells were pulsed with 0.5 μCi/well of [^3^H]thymidine for 18 h and harvested. [^3^H]Thymidine incorporation was determined in a scintillation counter (Topcount; Packard, Meridan, CT, USA).

IFN-γ production was measured in supernatant by ELISA (Pharmingen, San Diego, CA, USA) according to the manufacturer’s instructions.

IFN-γ ELISPOTs (Mabtech; San Diego, CA, USA) were done according to the manufacturer’s instructions. PBMCs obtained before and after vaccination were plated in 96-well plates at the concentration of 1 × 10^5^ cells per well with culture medium alone, with 1 × 10^5^ mature, patient’s tumor lysate-pulsed DC or with 10 μg/ml of lysate tumor only in the case of patients in wich DCs are not available. Spots quantification was performed using an automated ELISPOT reader (CTL, Aalen, Germany).

Changes in cytokines profiles after vaccination were evaluated in serum samples obtained before and after vaccination by Multiplex Bead Immunoassay Kit (Invitrogen, Carlsbad, CA, USA) for simultaneous quantitative determination of GM-CSF, IFN-α, IFN-γ, IL-1Rα, IL-1β, IL-2, IL-2R, IL-4, IL-5 IL-6, IL-7, IL-8, IL-10, IL-12, IL-13, IL-15, IL-17, IP-10, MCP-1, MIG, MIP-1α, MIP-1β, RANTES and TNF-α according with the manufacturer instructions and using Luminex^®^ xMAP^®^ system (Luminex, Austin, Texas).

### Evaluation of inflammatory infiltrate in tumor samples by flow cytometry

The obtained tumor biopsy was subjected to a disaggregation process using the GentleMACS dissociator (Miltenyi, Biotech, Germany) to obtain a single cell suspension. After washing twice, the cells were freeze following standard protocols. For flow cytometry analysis, an aliquot of cells was thawed and after 2 h at 37 °C, a panel of monoclonal antibodies was used to identify different cell subpopulations: percentage of tumor infiltrating lymphocytes [CD3 FITC (Miltenyi Biotec, clon BV264/56), CD4PB, CD8 BV510, Biolegend, clones RPA-T4 and RPA-T8 respectively], T naive (CD45RA+, CD62L+, CD27+): CD45RA PerCP Cy5.5 and CD27 APC were purchased by Biolegend, clones HI100 and 323 respectively and CD62L was purchased by BD Bioscience, clon DREG-56, T central memory (CD45RO+, CD62L+ CD27+): CD45RO PECy7 was provide by BD Bioscience, clon UCHL1, T effector memory (CD45RO+, CD62L−, CD27+) and T effector cells (CD45RO+, CD62L−, CD27−), CD69PE and HLA-DR FITC from Biolegend clones FN50 and L243 respectively were used as activation markers. We also studied the percentage of cell with an phenotype like myeloid derived suppressor cells (MDSC) (Lin−, CD33+, CD11b+, HLA-DR low/−): To define the Lin—population, a cocktail of PE-conjugated antibodies including CD3, CD16, CD19, CD20 and CD56 have been used, all of which were provided by Biolegend, clones OKT3, 368, SJ25C1, 2H7 and 5.1 H11 respectively; CD33 FITC, CD11b PerCP Cy5.5 and HLA-DR APC were purchased by Biolegend, clones HIM 3–4, ICR F44 and L243 respectively. Moreover in order to characterized monocytic or granulocytic MDSC we use CD14 BV421 clon M5E2 and CD15 BV510 clon W6D3 from Biolegend. Finally, we evaluate the expression of immunocheckpoint PD1 (Programmed cell death protein 1) (PD1 PerCP eFluor clone MIH-4 from Bioscience) in lymphocytes and expression of HLA-I (Human leukocyte antigen-I) in tumor cells (HLA-I FITC clone W6/32 from Biolegend). Briefly, cells were incubated with monoclonal antibodies conjugated to different fluorochromes during 15 min at room temperature and in the dark and then the cells were washed. Cells stained with isotype control antibody were used as negative control. Cells were acquired in FACSCALIBUR cytometer (Becton–Dickinson, Immunocytometry Systems, San Jose, CA, USA) and then analyzed using Flow Jo software.

### Statistical analysis

PFS and OS were analyzed with the Kaplan–Meier method.

For the in vitro experiments the software used for statistical analysis was GraphPad Prism. Changes in proliferation of post vaccines PBMC, IFN-γ producing cells number and cytokines profiles in serum after vaccination were analyzed with Wilcoxon test. Spearman correlation coefficient was used to investigate the relationship of immune response and OS.

## Results

### Patients

Thirty-two consecutive patients were reported in this paper (27 patients screened for the clinical trial and 5 treated as compassionate use before the clinical trial). One patient was not included in the trial because postoperative MRI showed a residual tumor volume of 3.4 cc^3^ and, as such, did not meet one of the inclusion criteria. The tumor location is described in Additional file 1, where representative preoperative and postoperative MRI images are also shown. Two patients suffered severe infectious complications (brain abscess and pneumonia, respectively) following surgery, and decided to withdraw their informed consent and discontinue participation in the trial before starting immunotherapy and standard chemotherapy. These patients were however included in the intention-to-treat analysis.

Median age was 61 years (mean 58.8, range 42–70). Karnofsky performance score (KPS) was 90–100 in 29%, 80 in 35.5% and 60–70 in 35.5% of cases. The MGMT was methylated in 45.2% of cases. In terms of RPA classes, 9.7% of the patients were in class III, 41.9% in class IV and 48.4% in class V. The mean preoperative tumor volume was 36.7 cc^3^. No residual tumor volume was observed in postoperative MRI in 25 of the cases (80.7%). Table 2 lists the basic characteristics of the patients.

### Feasibility

Enough tumor lysate and DCs were available in all cases to produce at least 6 vaccine doses. In all cases except one, steroid tapering could be performed within a few days after surgery, and the first vaccine was administered between days 21 and 29 after surgery (median 23). In the remaining case, the first vaccine was delayed until day 50.

### Safety and tolerability

Neither adverse events nor toxicity attributable to the immunotherapy were documented.

All severe adverse events (7.9% of total adverse events) were related to the standard therapy. Two patients (6%) had new deficits persisting 1 month after surgery, one patient had hemianopsia, and one patient had left hemiparesis. Two patients had neutropenia grade 3 (6%), and two thrombocytopenia grade 3 (6%). There were 2 cases of fatal bacterial pneumonia in vaccinated patients unrelated with the vaccine.

### Survival

**Fig. 2:**
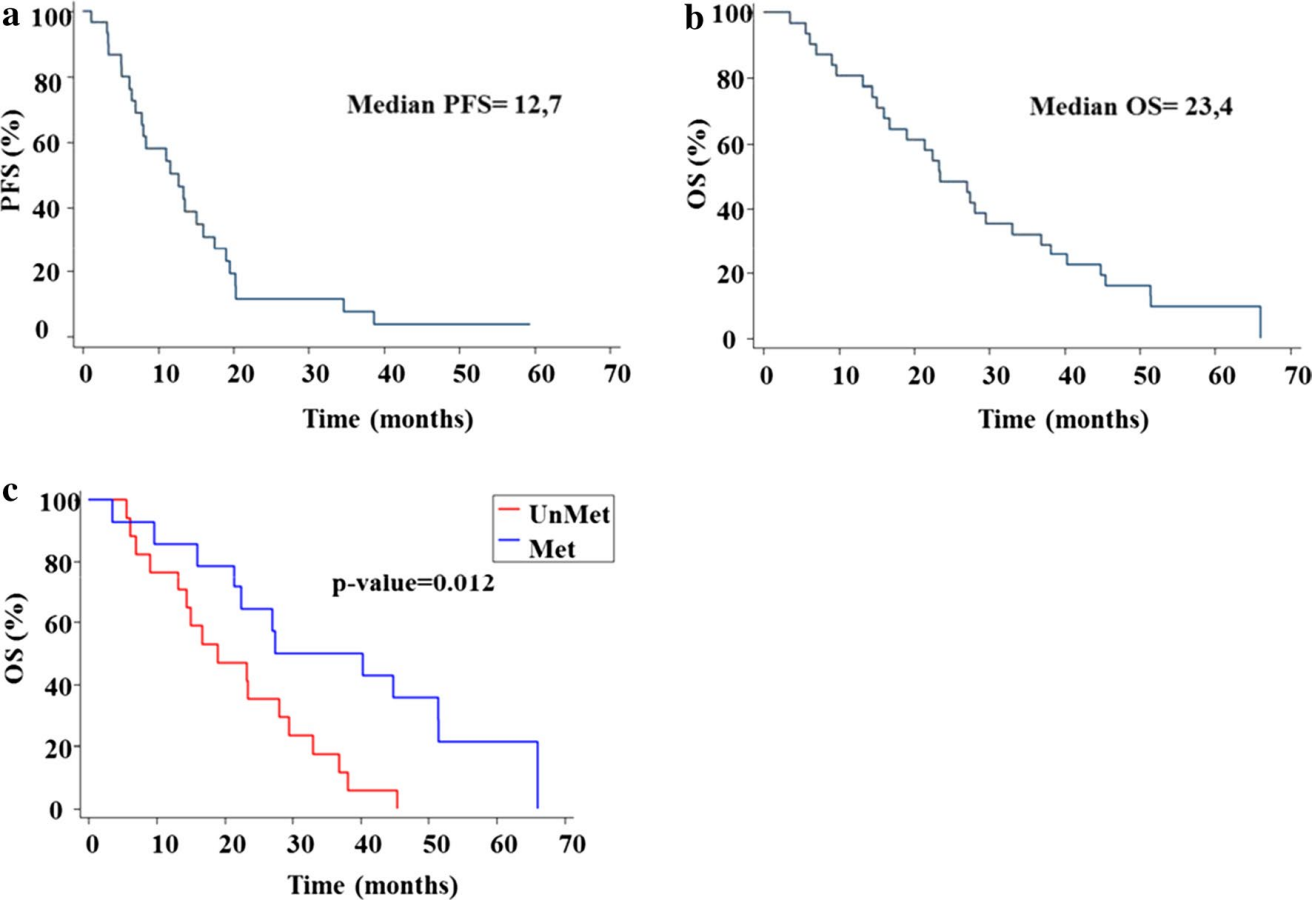
PFS and OS from patients treated with standard treatment plus dendritic cells vaccines. The patients were treated with standard treatment: radio-chemotherapy followed by chemotherapy of maintenance plus DC vaccines. PFS and OS were analysed with Kaplan–Meier method: **a** PFS in months from patients vaccinated; **b** OS in months from all patients, **c** OS in months from patients with MGMT unmethylated vs methylated

The median of PFS in all patients was 12.7 months (CI 95% 7–16) and the OS was 23.4 months (95% CI 16–33.1) (Fig. 2).

OS of patients with methylated MGMT promoter was statistically significant superior (Fig. 2c) to that of the patients with unmethylated MGMT promoter (median 27.4 versus 19 months; CI 95% 16–51.4 vs 9.1–29.5 respectively).

### Immune response

**Fig. 3:**
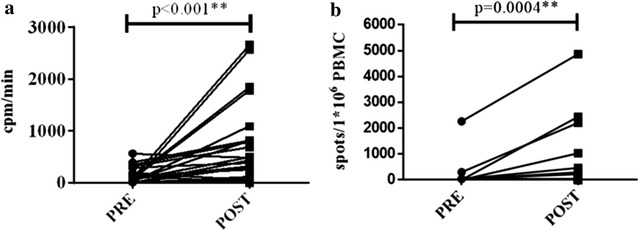
Tumor specific immune response in vaccinated patients. Blood samples were obtained before each vaccine. Mononuclear cells (PBMCs) before each vaccine were cryopreserved and thawed together to evaluate changes in proliferation of PBMC stimulated in presence of antigen (**a**) and number of IFN-γ producing cells by IFNγ enzyme-linked immunospot or ELISPOT after in vitro stimulation with pulsed DC (**b**)

An increase in proliferation of post vaccines PBMC after stimulation with tumor lysate-pulsed DCs or lysate tumor was detected in 11 of 27 patients who were tested. Moreover, when we analyzed all patients, we have found statistically significant differences between proliferation in samples before vaccines and samples obtained after receiving vaccines (p > 0.001) (Fig. 3).

With regard to cytokine production, we detected an increase in IFN-γ production (by ELISA or ELISPOT) in samples obtained after vaccines with respect to samples before vaccines in 8 of 25 patients. When we analyzed IFN-γ producing cells from all patients, we detect a statistically significant increase in the number of IFN-γ-producing cells in samples taken after vaccines with respect to samples collected before vaccines (p = 0.0004*) (Fig. 3). Changes in cytokines profiles after vaccination were evaluated in serum samples obtained before and after vaccination by Multiplex Bead Immunoassay Kit. In particular, 25 cytokines were quantified in serum samples obtained before and after vaccination. The concentrations of serum cytokines before and after vaccination are showed in Table 3. The results showed no statistically significant changes in cytokine profile in serum samples following vaccination with DC.

**Table 3:** Concentrations of serum cytokines

	Before vaccination	After vaccination
IL-1β	52.5 ± 33.5	53.7 ± 49.1
IL-10	321.6 ± 260.3	272.1 ± 249.3
IFN-α	70.9 ± 56.9	79.3 ± 64.8
IL-6	209.5 ± 280.6	160.4 ± 134
IL-12	134.2 ± 86.4	242.8 ± 581.7
RANTES	10,159.9 ± 820	9828 ± 1289.8
EOTAXIN	1490.8 ± 461.1	1412.4 ± 465.6
IL-13	229.8 ± 125.3	228.3 ± 112.4
IL-15	382.6 ± 68.6	402.8 ± 130.7
IL-17	194.2 ± 131	219 ± 152
MIP-1α	46.33 ± 17.8	76 ± 160
GM-CSF	139.1 ± 96.9	179.4 ± 264
MIP-1β	109.6 ± 48.1	100.4 ± 57.4
MCP-1	868.1 ± 637.6	717.1 ± 368.8
IL-5	112.7 ± 151.2	81.2 ± 77.8
IFN-γ	116.7 ± 82	108.3 ± 75.9
TNF-α	28.52 ± 42.3	31.1 ± 37.9
IL-1RA	218.7 ± 143.9	133.5 ± 71
IL-2	33.8 ± 25	27.4 ± 20.4
IL-7	366.5 ± 194.4	365.9 ± 194.9
IP-10	1846.1 ± 3590.6	690.4 ± 1170.4
IL-2R	296.9 ± 114.5	251 ± 55.6
MIG	272.7 ± 554.2	78.4 ± 61.8
IL-4	57.9 ± 58.2	44.7 ± 54.4
IL-8	182.7 ± 196.5	140.5 ± 67.5

Changes in cytokines profiles after vaccination were evaluated in 20 patients in serum samples obtained before and after vaccination by Multiplex Bead Immunoassay Kit (Invitrogen, Carlsbad, CA, USA) for simultaneous quantitative determination of 25 Human Cytokines. The median and standard deviation of all patients before and post vaccination for each cytokine are included in this table

No correlation was found between immune response detected (proliferation or IFN-γ producing cells) and OS (data not show).

### Study of the inflammatory infiltrate in tumor samples

In two patients, we had tumor sample from the initial biopsy and tumor sample from relapse. So, in these patients we were able to assess the effect of the treatment in inflammatory infiltrate.

GBM is a very necrotic tumor so the amount of live tissue was very limited in both patients (6.11% in patient 1 and 11.40% in patient 2 in the initial sample and 0.49 and 0.22% respectively in the relapse sample). In both cases, the number of living cells was very low (in patient 1 a mean of 897 per panel in the initial sample and 1698 in the relapse sample and in patient 2 a mean of 1536 per panel in the initial sample and 841 in the relapse sample). In patient 1 (Table 4) the percentage of CD3 was similar in the sample of diagnosis and in the relapse sample (7.1 vs 7.7% of living cells) and was similar to CD4 percentage (36.4 vs 37.7% of CD3+ cells) and CD8 percentage (56.1 vs 49.1% of CD3+ cells). In patient 2, we observed a slight increase in the percentage of CD3 in the sample of relapse (10.80 vs 19.6% of living cells). In this case, we can observe a slight increase in CD4 and decrease in CD8 in relapse samples regarding diagnosis (CD4: 17.1 vs 30.8% of CD3+ cells; CD8: 72.9 vs 62.8% of CD3+ cells). Detailed evaluation of lymphocyte populations before and after treatment in the 2 available samples is described in Table 4.

**Table 4:** Analysis of the inflammatory infiltrate in tumor samples

	Patient 1				Patient 2			
	Basal (%)		Relapse (%)		Basal (%)		Relapse (%)	
CD3	7.1		7.7		10.8		19.6	
CD4 (in CD3 subset)	36.4		37.7		17.1		30.8	
CD8 (in CD3 subset)	56.1		49.1		72.9		62.8	
MDSC-like phenotype cells	8		2		4.29		0.75	

	CD4+ cells		CD8+ cells		CD4+ cells		CD8+ cells	
	Basal (%)	Relapse (%)	Basal (%)	Relapse (%)	Basal (%)	Relapse (%)	Basal (%)	Relapse (%)
CD69	90.6	97.4	77	83.4	87.7	84.2	94.72	92
HLA-DR	71.85	59	57	46.48	56.1	56.17	37.1	50.29
Central memory	14.3	32.5	0	8.05	6.97	6.89	2.09	1.59
Efector memory	78.6	62.5	13.08	38.6	55.78	58.67	34.74	20.76
Effector	7.4	0	56.5	40.3	23.22	10.32	54.15	36.5
PD1	53.1	75	10.77	52	43.9	63.2	52.5	38.3

	MFI in Tumor cells		MFI in Tumor cells	
	Basal	Relapse	Basal	Relapse
HLA-expression	36,969	10,038	42,433	14,245

A single cell suspension were obtained from biopsy sample by a mechanical disaggregation process. After washing twice, the cells were freeze following standard protocols

For flow cytometry analysis, an aliquot of cells was thawed and after 2 h at 37°, a panel of monoclonal antibodies was used to identify different cell subpopulations. Results are expressed in percentage regarding total alive cells (CD3 and myeloid suppressor cells), and regarding CD3+ cells (CD4 and CD8). Activation markers (CD69 and HLA-DR) and a panel of markers to characterize different T cell population were evaluated in CD4 and CD8 positive cells. HLA-I expression (mean fluorescence intensity) were measure in tumor cells

In all cases, cells were incubated with monoclonal antibodies conjugated to fluorochromes during 15 min at room temperature and in the dark and then the cells were washed. Cells stained with isotype control antibody were used as negative control. Cells were acquired in FACSCALIBUR cytometer (Becton–Dickinson) and then analyzed using Flow Jo software

Among the data shown on the table, it is especially interesting that in both cases we observed an increased expression of PD-1 in lymphocyte from relapse sample with regard to the basal sample (in CD4+ and CD8+ cells in patient 1 and only in CD4+ cells in the patient 2). Also in both cases, we observed a decrease in cell with an MDSC-like phenotype in the relapse sample compared to basal samples (8 vs 2% in patient 1 and 4.29 vs 0.75% in patient 2). Finally, we have seen that in both cases there was a decrease in the expression of HLA-I (mean of fluorescence intensity) in tumor cells in the sample of relapse with regard to diagnosis sample.

## Discussion

Tumor-lysate pulsed DC vaccination has already been shown to be feasible and safe in previous trials. Moreover, some very long survival times have been reported, although efficacy is far from being proven as no randomized controlled trial has been published. Previous phase II trials showed an unusually long survival that probably would be due to their selective inclusion criteria (mostly young patients with good functional status) and exclusion criteria (patients with radiological progression or need for steroids after radiochemotherapy) [12–14, 18–20] (Table 5), which may introduce a selection bias that may affect the clinical results. As desired with this type of immunotherapeutic approach, our study was conducted on a highly selected population of GBM patients, i.e. those undergoing complete or near complete resection. In addition to other prognostic factors such as age, KPS (our cohort of patients included a representation of all RPA classes), the 23.4 month median OS observed in our trial could be in part related to the immunotherapeutic treatment.

**Table 5:** Relevant patients’ characteristics at the time of accrual in clinical trials on dendritic cell vaccination in glioblastoma multiforme

	Mean age	KPS (100/90/80/≤70)	RPA (class 3/4/5)	OS (months)
^a^Prins^8^	49.7	(7%; 77%; 20%; 7%)	(60%; 33%; 7%)^d^	35.9
^a^Ardon^9^	50.4	N/A	(12%/87%/0%)	24.0
^b^Sampson^25^	52.4	(39%; 39%; 22%; 0%)	(28%/72%/0%)^d^	26.0
^c^Phuphanich^26^	55.3	(12%; 62%; 12%; 12%)	(19%/69%/12%)^d^	38.4
Present data (intention-to-treat)	58.8	(6%; 23%; 36%; 36%)	(10%/42%/48%)	23.4

*KPS* Karnofsky performance status, *RPA* recursive partition analysis, *OS* overall survival, *N/A* not available

^a^It excludes patients with steroids after radiotherapy

^b^It excludes patients progressing after radiotherapy

^c^It excludes patients with more than 4 mg/day of dexamethasone

^d^RPA classes estimated based on provided data; in doubt, the highest level was assigned

Therefore, the only favorable factor embedded in our inclusion criteria was that we performed an extensive resection in all cases. In other words, we included only patients with potentially resectable tumors, which is a limitation of this immunotherapeutic approach. However, the effort to do an extensive resection was part of the study treatment, and only one case was excluded due to residual tumor. This fact is of importance because it excludes the presence of a selection bias in favor of small, superficial tumors. On the contrary, all patient candidates for resection surgery were enrolled, and a deliberate effort to carry out the maximum resection that was technically achievable was made. FGS and the intention to make a gross or near gross total resection were part of the treatment protocol. We have previously shown that, with experience in FGS, the objective of less than 1 cc^3^ residual tumor can be achieved in a majority of patients [8]. Similar results have been published with intraoperative MRI [21]. Such extensive resection can explain some of the observed benefit. Previous studies including patients with different extents of resection suggest that complete tumor removal might increase survival by approximately 4–5 months as compared with partial resection [22–24]. According to the GBM survival calculator based on the MDACC cohort [25], the expected median OS for our group of patients is 15.8 months. It is therefore possible that immunotherapy, in combinations with such wide surgical resections, might have played a role in our results.

The percentage of patients with methylated MGMT promoter was 45.2%, as usually described in general population of GBM patients. Thus, no apparent bias regarding the status of MGMT promoter appears to explain, solely by itself, the outcome times obtained in our study.

Other critical point is the design of the vaccine schedule. Compared to previous DCs vaccination works in GBM, we began up-front immunotherapy prior to radiotherapy. During the usual 4-week interval between surgery and radio-chemotherapy, there is sufficient time to wane the patient off steroids, manufacture the vaccine and administer its first dose. We do not necessarily expect therapeutic benefit from this dose during the subsequent radio-chemotherapy period, but we reasoned that it might help to prime the immune system and to allow a faster immune response build-up after subsequent doses. Moreover, the effort to keep vaccinating patients even after progression could be important for the improvement of the OS.

The lack of benefit in PFS and correlation between overall survival and immune response deserve a further comment. While PFS results were far less compelling than those concerning OS, the time between the day of progression and death is much longer than usual in GBM. Three different factors can contribute to explain this paradox. First, the definition of progression in GBM is not clear; pseudoprogression has been increasingly recognized, and its incidence could be even greater during or after immunotherapy. In other words, we may have inadvertently misinterpreted as radiological progressions at least some cases of radiologically indistinguishable local immune reactions. This is a common problem for all the immunotherapy trials presently studied. In these trials, the overall survival could be the only objective parameter to measure clinical efficacy [26] or it may be more appropriate to use iRANO guidelines to evaluate responses to immunotherapy treatments [27]. Second, the immune effect of vaccination may not develop rapidly enough to avoid progression in some cases, but it could still help to delay disease progression and to maximize the benefit of second-line therapies such as subsequent surgery or new chemotherapy. This type of effect has already been reported for some chemo-immunotherapy combinations [28, 29]. Third, the discrepancy between PFS and OS could be attributed to the effect of a second therapy. Should this be the case, we would be introducing an important caveat, as benefit from vaccination might not be as substantial as we think. In our patients, the most used second-line agent at progression was bevacizumab, a drug with some recognized activity in GBM. However, this does not explain most of the benefit because in our series, second PFS as well as OS measured from the day of first progression are clearly longer than the corresponding survivals obtained with bevacizumab [30]. Additionally, the recent large randomized trials AVAGLIO and RTOG-0825 have been unable to show a benefit for bevacizumab in OS, which was approximately 16 months. We believe that the correct interpretation of all these data is that DC vaccination takes time to stimulate a specific immune response, but the effect is prolonged and can be synergistic with that of subsequent therapies.

Regarding the immune response, although we detected an increase in both proliferation and number of IFN-γ producing cells after antigen stimulation of PBMC obtained pre and post vaccination, we found no correlation between immune response and survival. Earlier studies with DC vaccines have confirmed immune activation against tumor and suggest an improvement in survival, but do not uniformly show a correlation between survival and immune response [14, 31–34]. There are many reasons that can explain this. There are many different assays to detect and quantify antigen-specific immune response both in vivo and in vitro, but all of these strategies must be optimized and validated.

In our case, we performed the study of the immune response in frozen samples that had been extracted between 1 and 3 months after the administration of the vaccines, because the blood extraction was done prior to the administration of a new vaccine dose in order to avoid the patient from having more visits than those planned for the treatment. Perhaps to optimize the immune response detection it would have been better to extract the samples within 7–10 days after administration of the vaccines.

Another relevant point is the type of antigen used to pulse DC. When peptide or defined antigen are used, it is easier to measure the immune response against these known antigens (e.g. use of tetramers or TCR sequencing), but in our case we have used tumor-lysed pulsed DCs vaccine and measuring the response to lysates is much more difficult because immunogenic targets may be highly diluted.

Therefore, in this work, both the samples used and the tests performed to measure immune response have several limitations that can affect the results obtained. These limitations should be solved for the monitoring of the immune response in future clinical trials.

On the other hand, as has been previously published by other authors (31) even if these assays could be validated and standardized, it is possible that the subtle and complex immunologic shifts triggered by immunotherapies cannot be detected.

Finally, it should not be forgotten that in vitro assays may not reflect what actually happens in vivo, since the immunosuppressive environment characteristic of tumors can render the response ineffective. This is another reason why there could be no correlation between the in vitro immune response and clinical response.

In addition to in vitro immune response studies, in two patients, we have analyzed the inflammatory infiltrate in tumor samples obtained at diagnosis and at relapse. In both patients there are a decrease in the percentage of cell with an MDSC-like phenotype in relapse samples regarding basal samples. This finding is not surprising since there are already some reports that relate concomitant treatment of radiotherapy and TMZ with decreasing of MDSC. TMZ can lead to decrease CCL2 production by glioma cells [35]. CCL2, among others functions, is associated with recruitment of immunosuppressive leukocytes, such as MDSC and regularory T-cells (Tregs) [35, 36]. Moreover, CCL2 stimulates monocytes to migrate to the tumor, and there they are converted in MDSC and immunosuppressive tumor-associated macrophages and facilitate the tumor growth. Decrease of CCL2 can be empaires with reduction of the infiltration of MDSC and tumor-associated macrophages (TAM) into the glioma site. In our case, the decrease of myeloid suppressor cells in the tumor could favor the effect of vaccines.

Moreover, in these patients, we could detect tumor infiltrating lymphocytes in the initial and in the relapse sample. The most relevant data is that these cells express a high percentage of activation markers (CD69 and HLA-DR), suggesting that there is an activation of the immune system. However, we have seen that it is possible that the tumor is developing immune escape mechanisms that cause the immune response was less effective than it could. In the two patients studied, we found a decrease in expression of HLA-I molecules (in terms of mean of fluorescence intensity) on tumor cells in samples after vaccines respect to baseline. We have also seen also in both cases an increase in expression of PD-1 in lymphocytes obtained from tumor samples at relapse with respect to samples of the initial tumor. This could mean that although an immune response is induced by the vaccine (PD1 is upregulated after antigen stimulation through TCR), the immune system by itself develops control mechanisms that contribute to inhibit the generated immune response. Recent work showed that PD-1 could be a biomarker for intratumoral, tumor specific CD8+ lymphocytes in melanoma [37] and this could be valid for GBM due to the high percentage of CD8+ cells expressing PD-1 in ours samples, especially in tumors relapse. To resolve this hypothesis further experiments should be performed. Also, this could be important to design strategies for enhance the effect of vaccines. The combination of vaccines and immunocheck point inhibitors could be a very interesting strategy to improve the results obtained so far in clinical trials with vaccines. In this sense, there are already launched several clinical trials exploring this possibility.

Our results confirm that DCs vaccination is feasible—with first vaccine administered even before starting radiotherapy—and safe, and seem to suggest that this approach could contribute, at least in part, to some survival benefit in this selected population of GBM patients undergoing complete or near complete resection. The latter point warrants a confirmatory multicenter randomized clinical trial. Additionally, further studies investigating the combination of CDs vaccination with other immunotherapy strategies are needed.

## Conclusion

The addition of tumor lysate-pulsed autologous DCs vaccination to maximal safe resection followed by radiotherapy and concomitant and adjuvant temozolomide is feasible and safe. Its potential benefit in survival in such a selected population still needs to be confirmed in a randomized trial.

## Additional file

**Additional file 1: Appendix 1.** Tumor location & preoperative and postoperative MRI images.

## Abbreviations

5-ALA: 5-aminolevulinic
CR: complete radiological resection
DC: dendritic cell
ELISA: enzyme-linked immunosorbent assay
ELISPOT: enzyme-linked immunospot assay
FGS: fluorescence-guided surgery
GBM: glioblastoma multiforme
GM-CSF: granulocyte-macrophage colony-stimulating factor
IFN-α: interferon-α
IFN-γ: interferon-γ
IL-4: interleukin-4
MDSC: myeloid-derived suppressor cells
MGMT: O6-methylguanine-DNA-methyltransferase
MRI: magnetic resonance imaging
OS: overall survival
PBMC: peripheral blood mononuclear cells
PFS: progression free survival
RPA: recursive partition analysis
RPMI: Roswell Park Memorial Institute medium
TMZ: temozolomide
TNF-α: tumor necrosis factor-α

## References

[1] Stupp R, Mason WP, van den Bent MJ, Weller M, Fisher B, Taphoorn MJ (2005). Radiotherapy plus concomitant and adjuvant temozolomide for glioblastoma. N Engl J Med.

[2] Chinot OL, Wick W, Mason W, Henriksson R, Saran F, Nishikawa R (2014). Bevacizumab plus radiotherapy–temozolomide for newly diagnosed glioblastoma. N Engl J Med.

[3] Gilbert MR, Dignam JJ, Armstrong TS, Wefel JS, Blumenthal DT, Vogelbaum MA (2014). A randomized trial of bevacizumab for newly diagnosed glioblastoma. N Engl J Med.

[4] Li J, Wang M, Won M, Shaw EG, Coughlin C, Curran WJ (2011). Validation and simplification of the Radiation Therapy Oncology Group recursive partitioning analysis classification for glioblastoma. Int J Radiat Oncol Biol Phys.

[5] Stupp R, Hegi ME, Mason WP, van den Bent MJ, Taphoorn MJ, Janzer RC (2009). Effects of radiotherapy with concomitant and adjuvant temozolomide versus radiotherapy alone on survival in glioblastoma in a randomised phase III study: 5-year analysis of the EORTC-NCIC trial. Lancet Oncol.

[6] Stummer W, Pichlmeier U, Meinel T, Wiestler OD, Zanella F, Reulen HJ, ALA Glioma Study Group (2006). Fluorescence-guided surgery with 5-aminolevulinic acid for resection of malignant glioma: a randomised controlled multicentre phase III trial. Lancet Oncol.

[7] Sanai N, Berger MS (2008). Glioma extent of resection and its impact on patient outcome. Neurosurgery.

[8] Diez Valle R, Tejada Solis S, Idoate Gastearena MA, Garcia de Eulate R, Dominguez Echavarri P, Aristu Mendiroz J (2011). Surgery guided by 5-aminolevulinic fluorescence in glioblastoma: volumetric analysis of extent of resection in single-center experience. J Neurooncol.

[9] Della Puppa A, De Pellegrin S, d’Avella E, Gioffre G, Rossetto M, Gerardi A (2013). 5-aminolevulinic acid (5-ALA) fluorescence guided surgery of high-grade gliomas in eloquent areas assisted by functional mapping. Our experience and review of the literature. Acta Neurochir (Wien).

[10] Schucht P, Beck J, Abu-Isa J, Andereggen L, Murek M, Seidel K (2012). Gross total resection rates in contemporary glioblastoma surgery: results of an institutional protocol combining 5-aminolevulinic acid intraoperative fluorescence imaging and brain mapping. Neurosurgery.

[11] Stummer W, van den Bent MJ, Westphal M (2011). Cytoreductive surgery of glioblastoma as the key to successful adjuvant therapies: new arguments in an old discussion. Acta Neurochir (Wien).

[12] Prins RM, Soto H, Konkankit V, Odesa SK, Eskin A, Yong WH (2011). Gene expression profile correlates with T-cell infiltration and relative survival in glioblastoma patients vaccinated with dendritic cell immunotherapy. Clin Cancer Res.

[13] Ardon H, Van Gool S, Lopes IS, Maes W, Sciot R, Wilms G (2010). Integration of autologous dendritic cell-based immunotherapy in the primary treatment for patients with newly diagnosed glioblastoma multiforme: a pilot study. J Neurooncol.

[14] Wheeler CJ, Black KL, Liu G, Mazer M, Zhang XX, Pepkowitz S (2008). Vaccination elicits correlated immune and clinical responses in glioblastoma multiforme patients. Cancer Res.

[15] Louis D, Ohgaki H, Wiestler O, Cavenee W, Burger P, Jouvet A (2007). The 2007 WHO classification of tumours of the central nervoussystem. Acta Neuropathol.

[16] Valle RD, de Cerio AL, Inoges S, Tejada S, Pastor F, Villanueva H (2012). Dendritic cell vaccination in glioblastoma after fluorescence-guided resection. World J Clin Oncol.

[17] Macdonald DR, Cascino TL, Schold SC, Cairncross JG (1990). Response criteria for phase II studies of supratentorial malignant glioma. J Clin Oncol.

[18] Liau LM, Prins RM, Kiertscher SM, Odesa SK, Kremen TJ, Giovannone AJ (2005). Dendritic cell vaccination in glioblastoma patients induces systemic and intracranial T-cell responses modulated by the local central nervous system tumor microenvironment. Clin Cancer Res.

[19] Sampson JH, Heimberger AB, Archer GE, Aldape KD, Friedman AH, Friedman HS (2010). Immunologic escape after prolonged progression-free survival with epidermal growth factor receptor variant III peptide vaccination in patients with newly diagnosed glioblastoma. J Clin Oncol.

[20] Phuphanich S, Wheeler CJ, Rudnick JD, Mazer M, Wang H, Nuno MA (2013). Phase I trial of a multi-epitope-pulsed dendritic cell vaccine for patients with newly diagnosed glioblastoma. Cancer Immunol Immunother.

[21] Hatiboglu MA, Weinberg JS, Suki D, Rao G, Prabhu SS, Shah K (2009). Impact of intraoperative high-field magnetic resonance imaging guidance on glioma surgery: a prospective volumetric analysis. Neurosurgery..

[22] Lacroix M, Abi-Said D, Fourney DR, Gokaslan ZL, Shi W, DeMonte F (2001). A multivariate analysis of 416 patients with glioblastoma multiforme: prognosis, extent of resection, and survival. J Neurosurg.

[23] Sanai N, Polley MY, McDermott MW, Parsa AT, Berger MS (2011). An extent of resection threshold for newly diagnosed glioblastomas. J Neurosurg.

[24] Stummer W, Reulen HJ, Meinel T, Pichlmeier U, Schumacher W, Tonn JC (2008). Extent of resection and survival in glioblastoma multiforme: identification of and adjustment for bias. Neurosurgery..

[25] Marko NF, Weil RJ, Schroeder JL, Lang FF, Suki D, Sawaya RE (2014). Extent of resection of glioblastoma revisited: personalized survival modeling facilitates more accurate survival prediction and supports a maximum-safe-resection approach to surgery. J Clin Oncol.

[26] Palucka K, Banchereau J (2012). Cancer immunotherapy via dendritic cells. Nat Rev Cancer.

[27] Okada H, Weller M, Huang R, Finocchiaro G, Gilbert MR, Wick W (2015). Immunotherapy response assessment in neuro-oncology: a report of the RANO working group. Lancet Oncol.

[28] Wheeler CJ, Das A, Liu G, Yu JS, Black KL (2004). Clinical responsiveness of glioblastoma multiforme to chemotherapy after vaccination. Clin Cancer Res.

[29] Finn OJ (2008). Cancer immunology. N Engl J Med.

[30] Chamberlain M (2011). Bevacizumab for the treatment of recurrent glioblastoma. Clin Med Insights.

[31] Fadul CE, Fisher JL, Hampton TH, Lallana EC, Li Z, Gui J (2011). Immune response in patients with newly diagnosed glioblastoma multiforme treated with intranodal autologous tumor lysate-dendritic cell vaccination after radiation chemotherapy. J Immunother..

[32] Yamanaka R, Homma J, Yajima N, Tsuchiya N, Sano M, Kobayashi T (2005). Clinical evaluation of dendritic cell vaccination for patients with recurrent glioma: results of a clinical phase I/II trial. Clin Cancer Res.

[33] De Vleeschouwer S, Fieuws S, Rutkowski S, Van Calenbergh F, Van Loon J, Goffin J (2008). Post-operative adjuvant dendritic cell-based immunotherapy in patients with relapsed glioblastoma multiforme. Clin Cancer Res.

[34] Yu JS, Liu G, Ying H, Yong WH, Black KL, Wheeler CJ (2004). Vaccination with tumor lysate-pulsed dendritic cells elicits antigen-specific, cytotoxic T-cells in patients with malignant glioma. Cancer Res.

[35] Huang B, Lei Z, Zhao J, Gong W, Liu J, Chen Z (2007). CCL2/CCR2 pathway mediates recruitment of myeloid suppressor cells to cancers. Cancer Lett.

[36] Sengupta Sadhak, Marrinan Jaclyn, Frishman Caroline, Sampath Prakash (2012). Impact of temozolomide on immune response during malignant glioma chemotherapy. Clin Dev Immunol.

[37] Gros A, Robbins PF, Yao X, Li YF, Turcotte S, Tran E (2014). PD-1 identifies the patient-specific CD8+ tumor-reactive repertoire infiltrating human tumors. J Clin Invest.

