# Supplementary material for: A phase II trial of autologous dendritic cell vaccination and radiochemotherapy following fluorescence-guided surgery in newly diagnosed glioblastoma patients
Source: J Transl Med. 2017 May 12;15:104. doi: 10.1186/s12967-017-1202-z (PMC5427614; doi:10.1186/s12967-017-1202-z)

APPENDIX 1. Tumor location & Preoperative and postoperative MRI images.

The tumor location and the correspondent pre- and postoperative T1WI+gadolinium MRI images are described for each patient, according to the following schema:

PATIENT NO.<br>Brief description of tumor location.<br>MRI images:<br>Left: Preoperative MRI. T1WI + GADO<br>Right: Early postoperative MRI. T1WI + GADO

PATIENT 1.
Right frontal lobe with mild corpus callosum involvement.

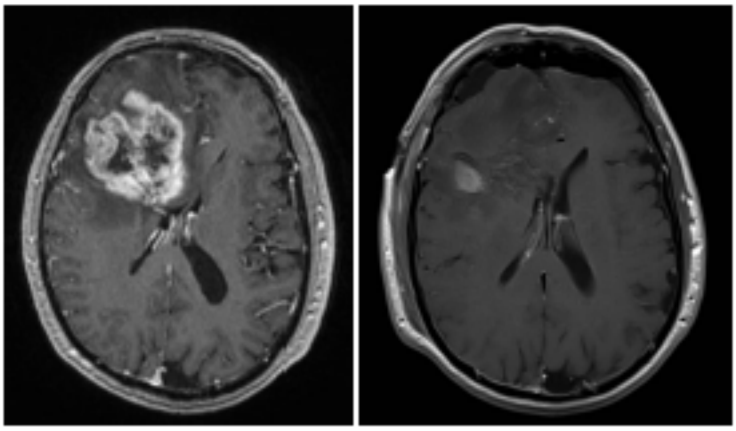

PATIENT 2.
Left temporal lobe with subependymal involvement.

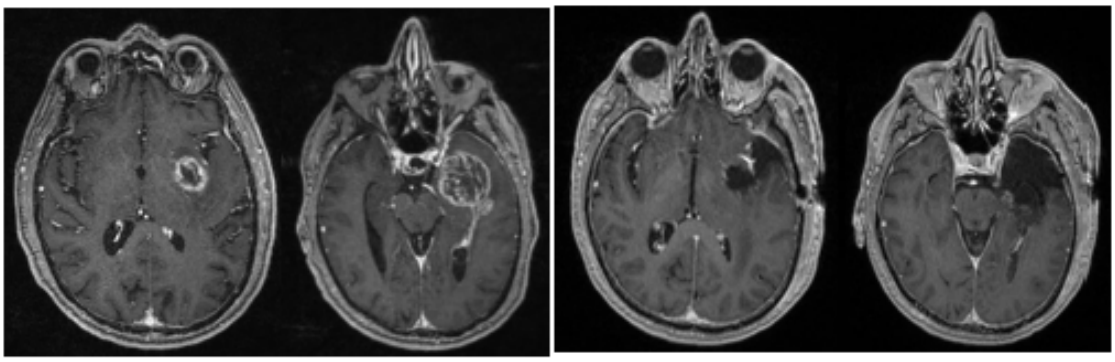

PATIENT 3.
Left temporal lobe.

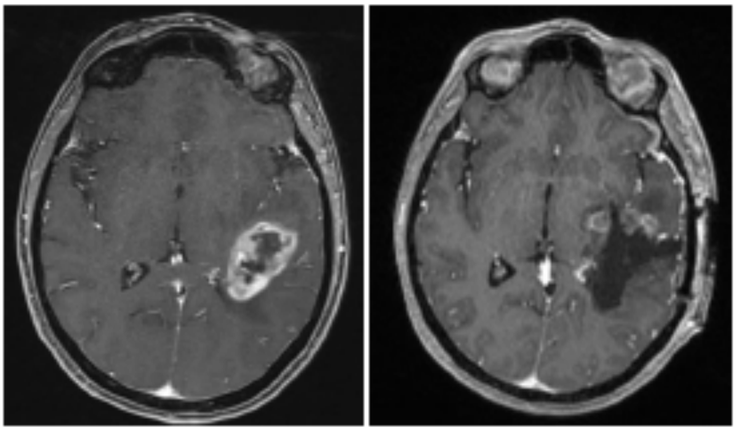

PATIENT 4.
Right frontal lobe.

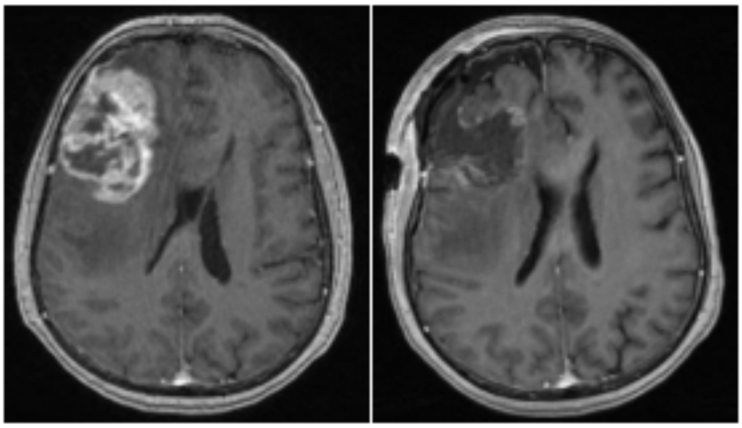

PATIENT 5.
Right frontal lobe.

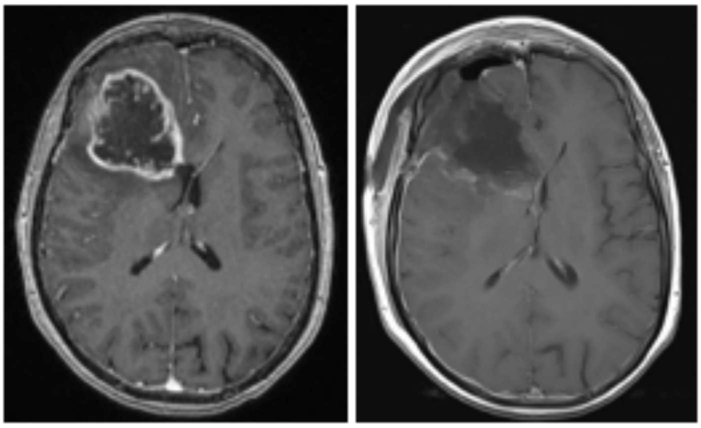

PATIENT 6.
Right temporal lobe.

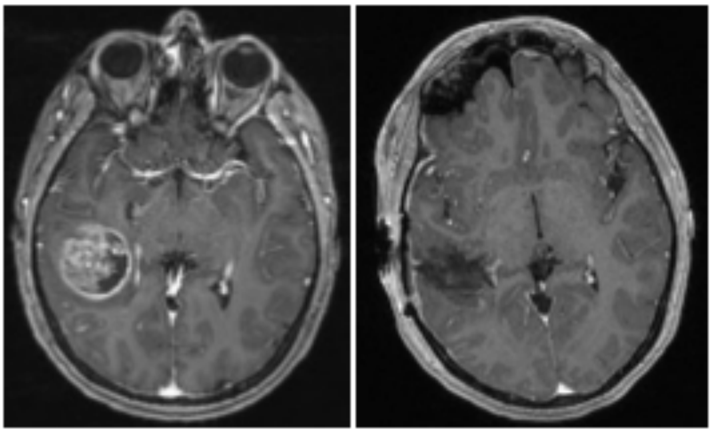

PATIENT 7.
Right medial temporal lobe with subependymal involvement.

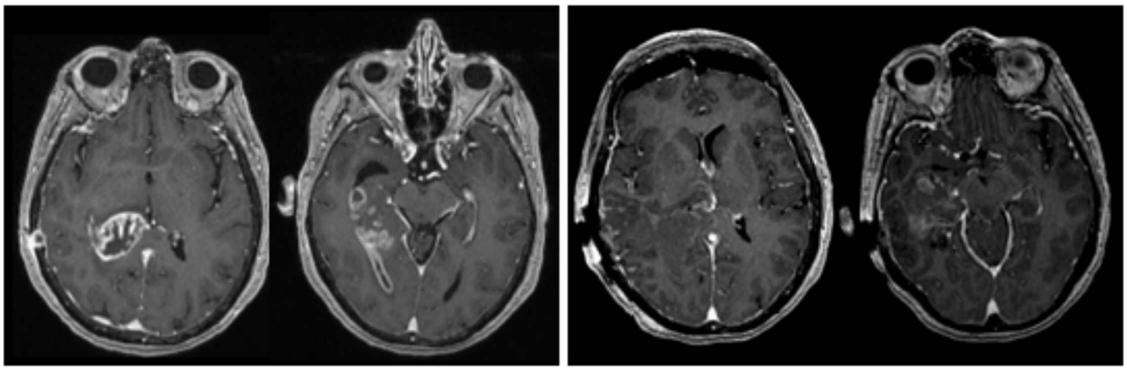

PATIENT 8.
Right temporal lobe.

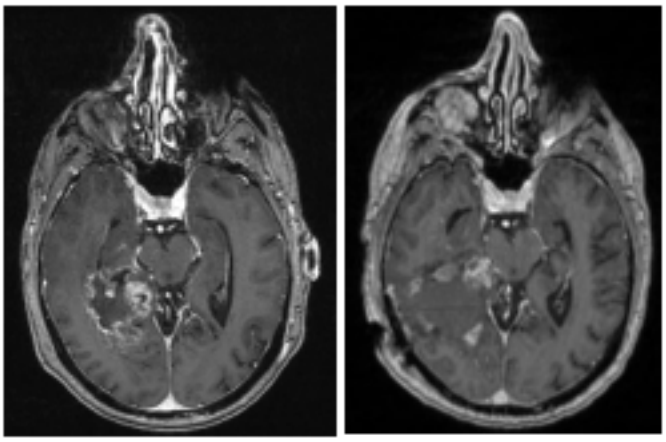

PATIENT 9.
Right frontal lobe with basal ganglia involvement.

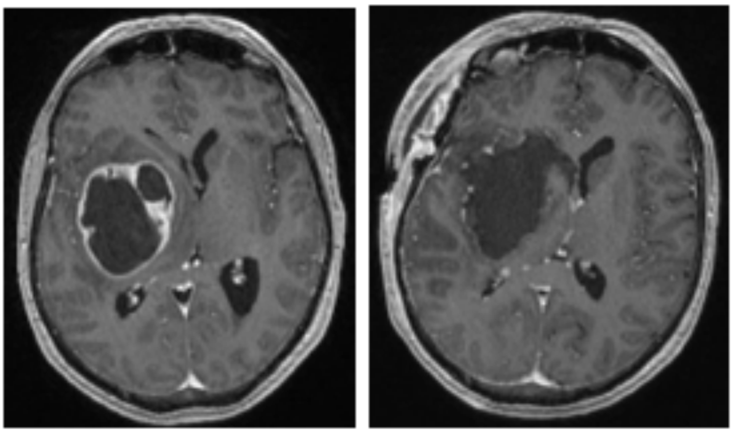

PATIENT 10.
Left temporo-occipital lobes.

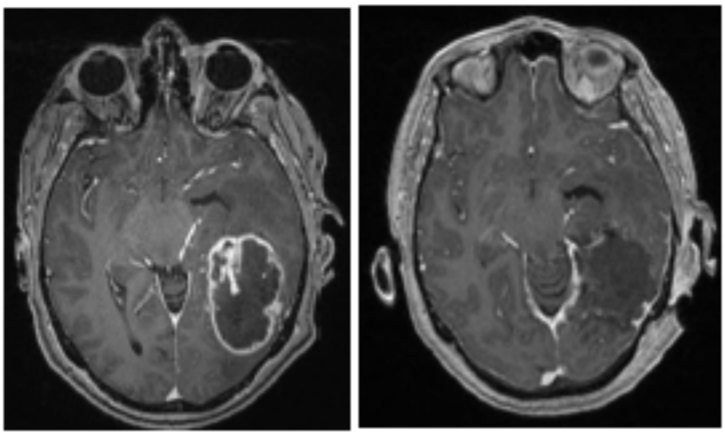

PATIENT 11.
Left parieto-occipital lobes.

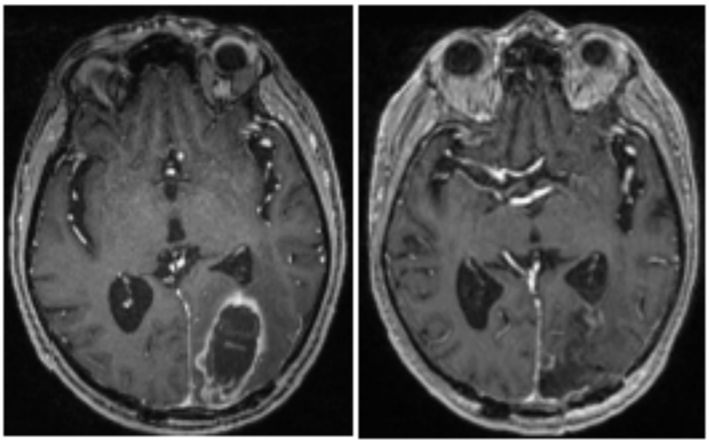

PATIENT 12.
Left temporal lobe.

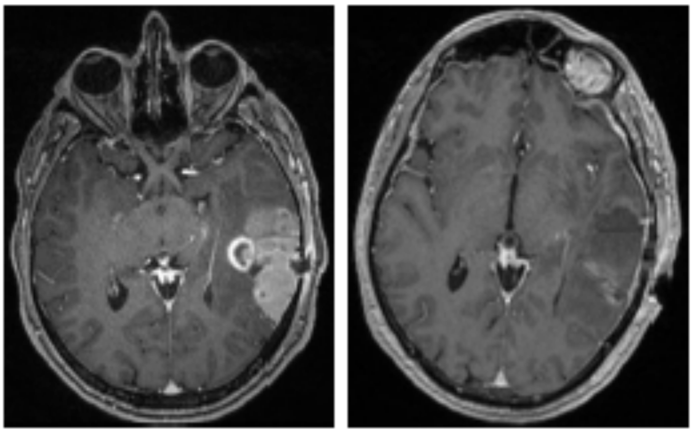

PATIENT 13.
Bilateral fronto-callosal location.

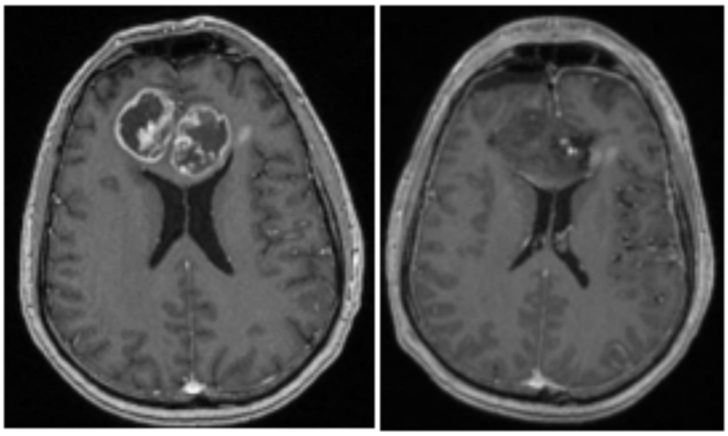

PATIENT 14.
Right parietal lobe.

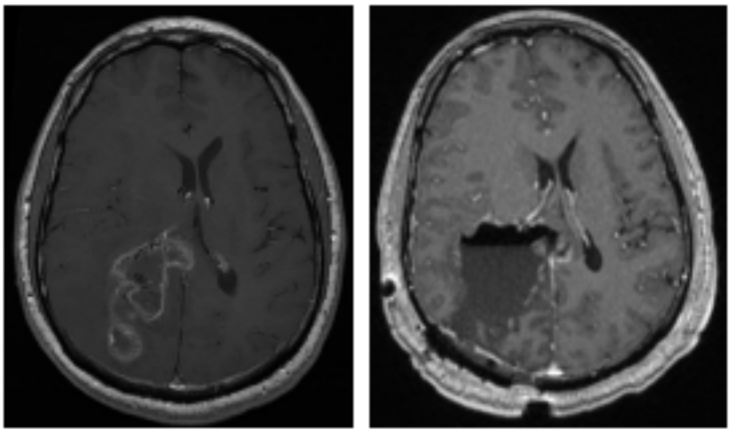

PATIENT 15.
Right frontal lobe.

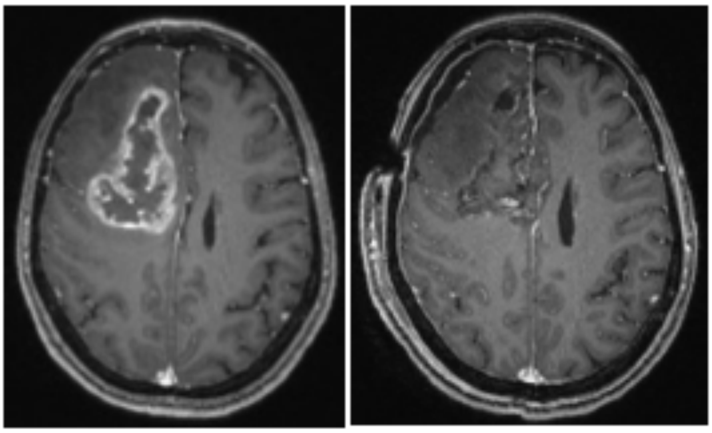

PATIENT 16.
Right temporo-parietal lobes.

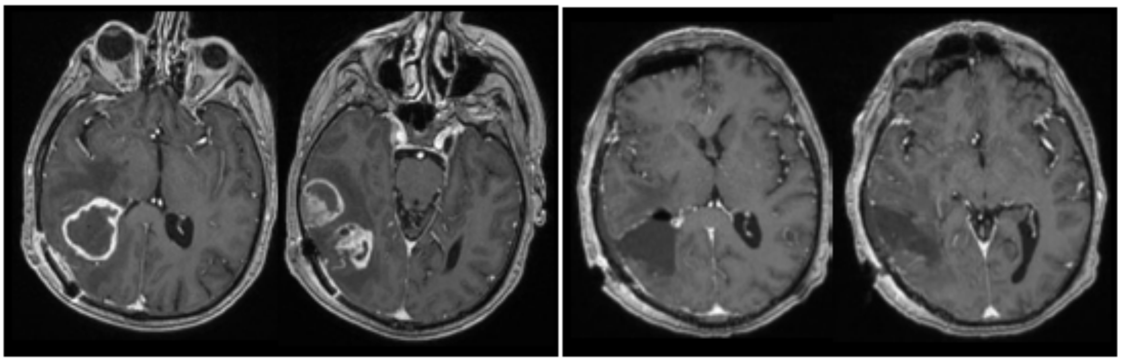

PATIENT 17.
Right parietal lobe.

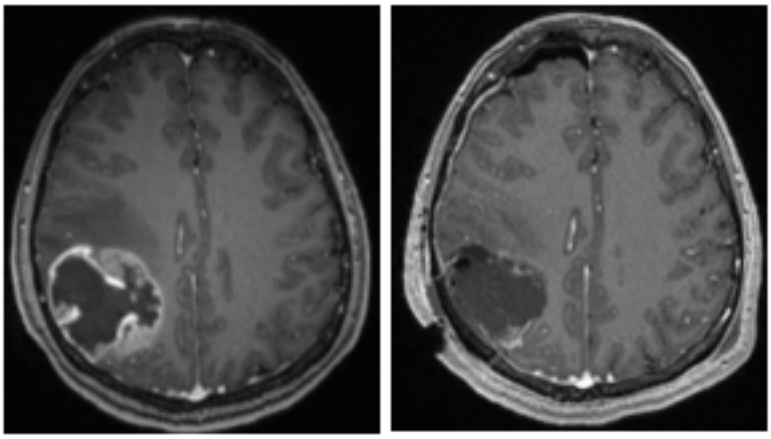

PATIENT 18.
Left medial frontoparietal lobes.

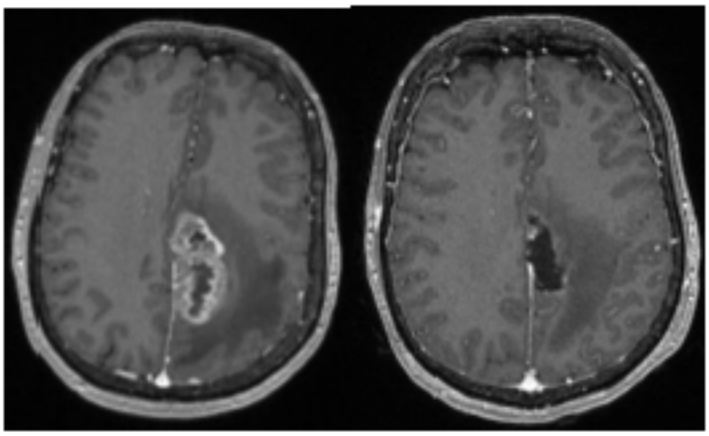

PATIENT 19.
Left temporo-occipital lobes.

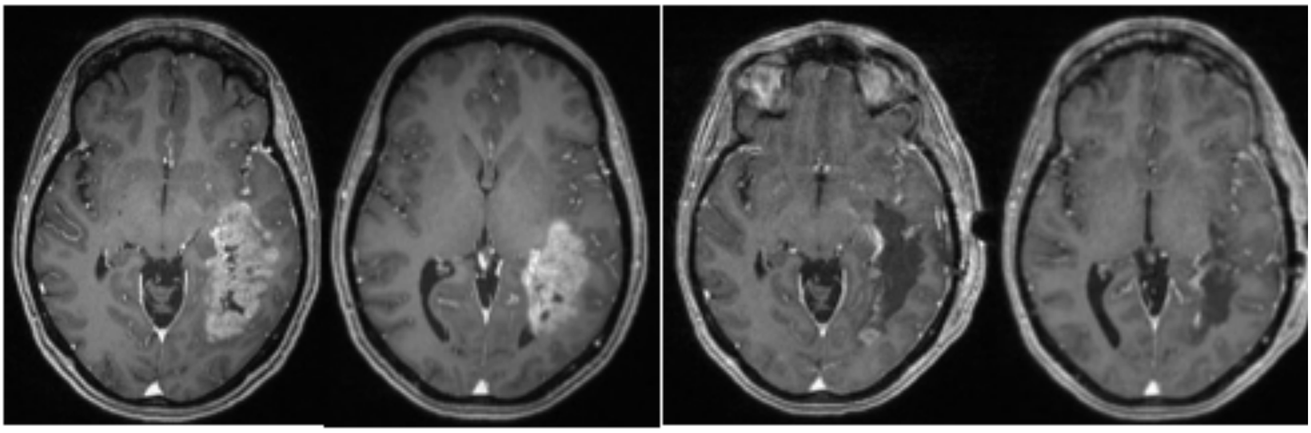

PATIENT 20.
Left parieto-occipital lobes.

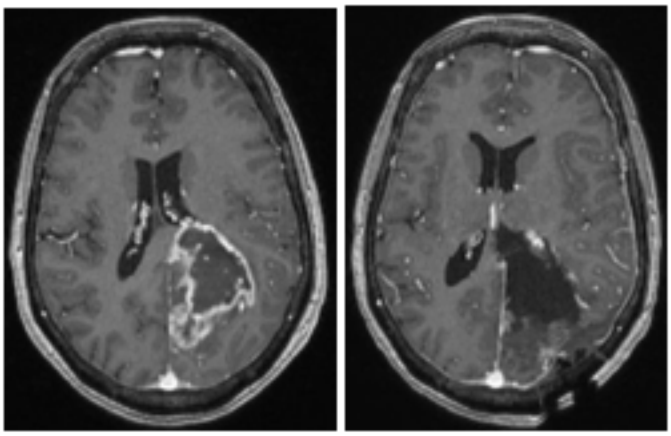

PATIENT 21.
Right frontal operculum área.

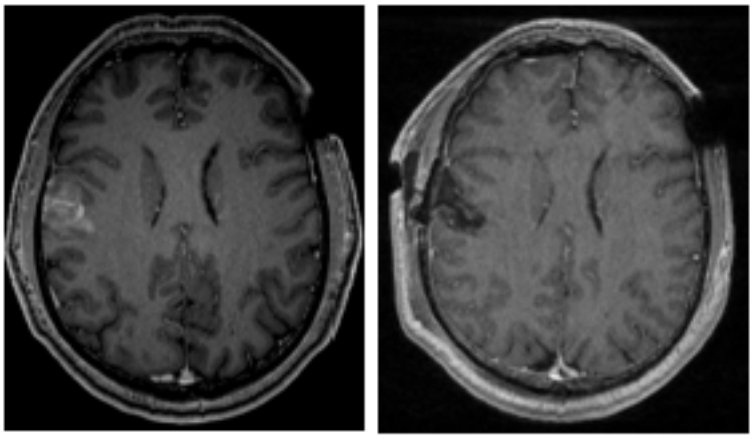

PATIENT 22.
Right fronto-temporal lobes.

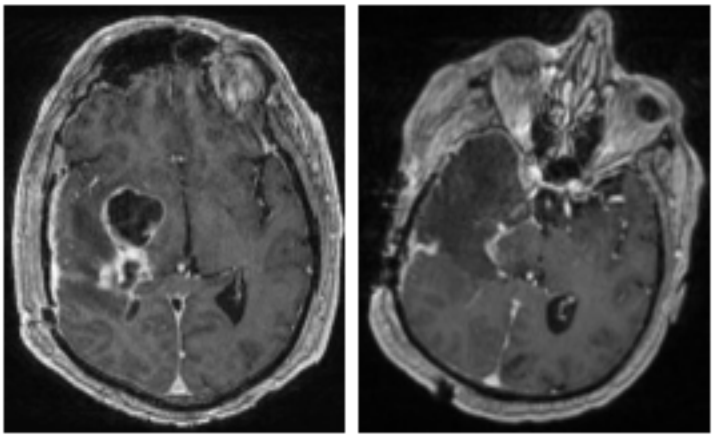

PATIENT 23.
Right temporo-parietal lobes.

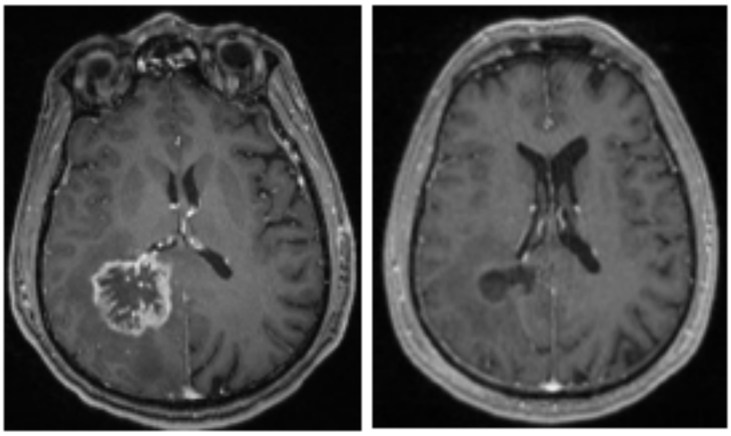

PATIENT 24.
Patient not included - screening failure (residual tumor > 1cc).

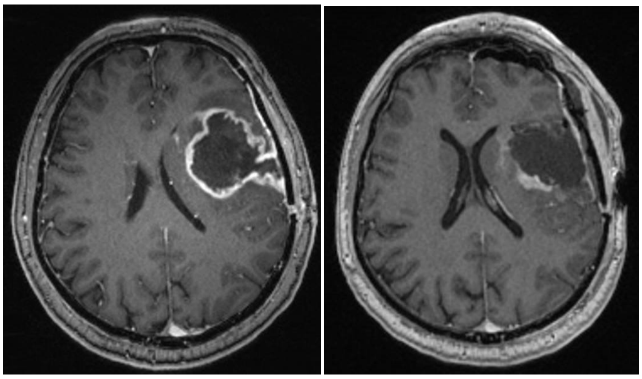

PATIENT 25.
Right frontal lobe.

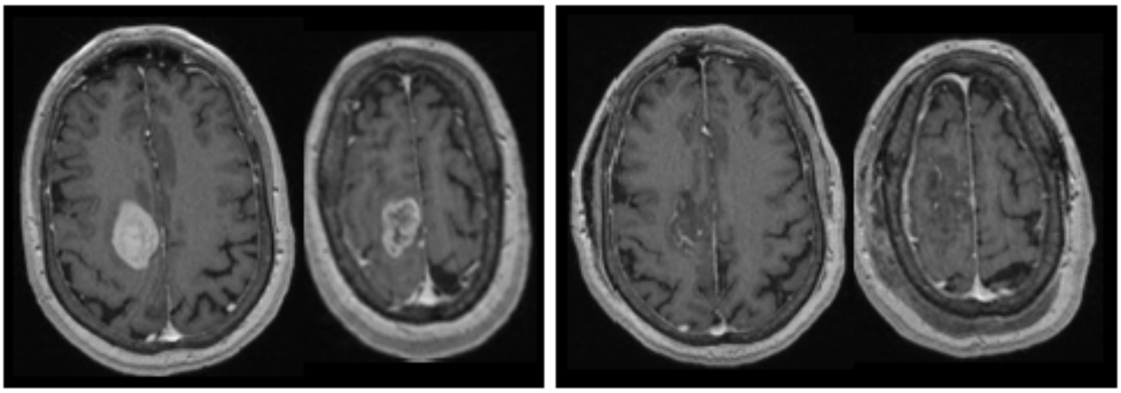

PATIENT 26.
Left temporal lobe.

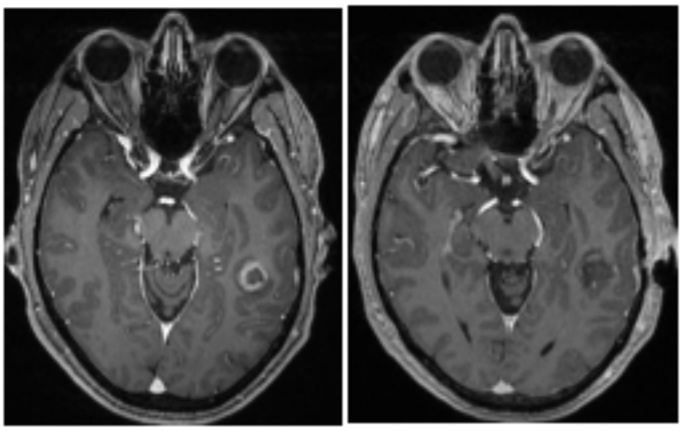

PATIENT 27.
Left temporal lobe.

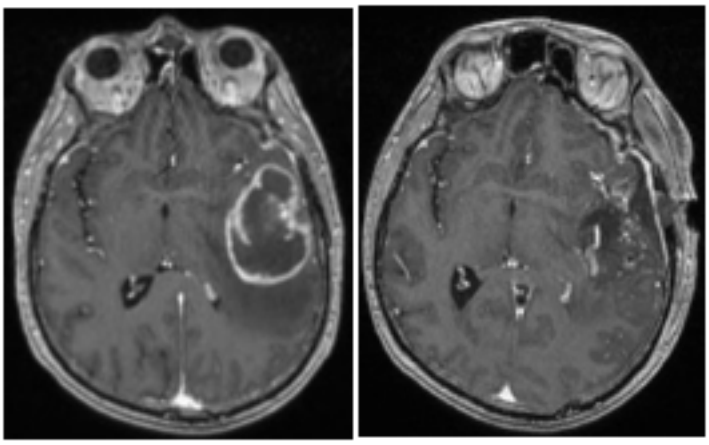

PATIENT 28.
Left fronto-parietal lobes.

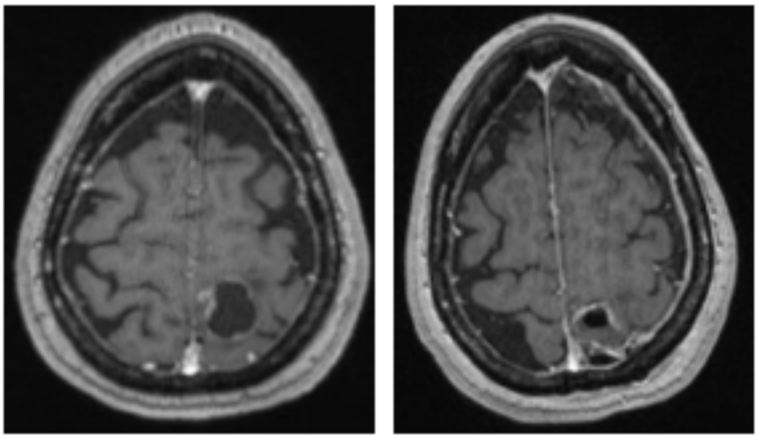

PATIENT 29.
Left parietal lobe.

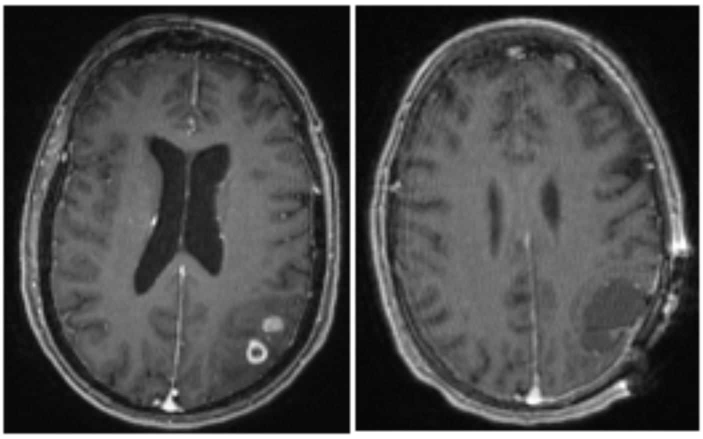

PATIENT 30.
Left frontal lobe.

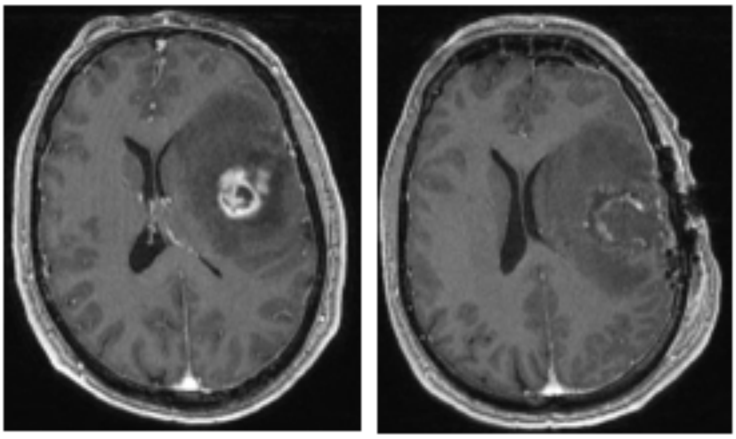

PATIENT 31.
Right frontal lobe with corpus callosum involvement.
Pre-operative MRI not available.

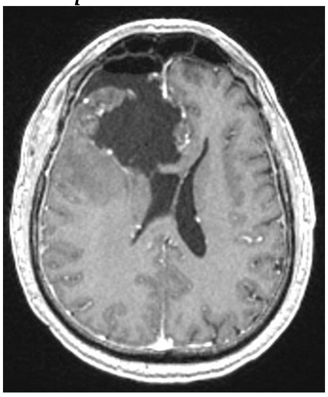

PATIENT 32.
Left parieto-occipital lobes.

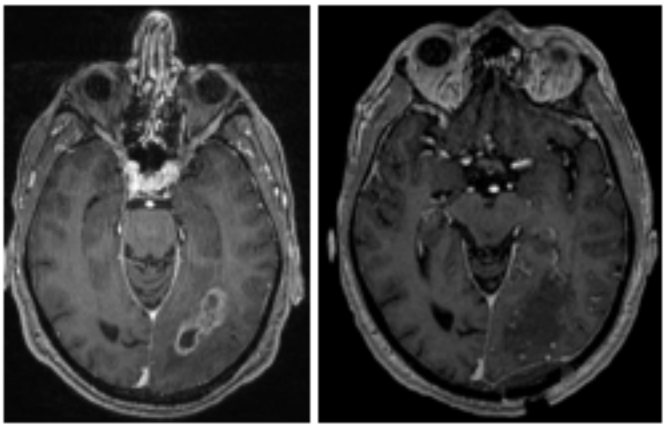

Supplement: Supplementary file 1 — Additional file 1: Appendix 1. Tumor location & preoperative and postoperative MRI images. [file 12967_2017_1202_MOESM1_ESM.pdf]
